# Sonographic Characteristics of Papillary Thyroid Carcinoma With Coexistent Hashimoto’s Thyroiditis in the Preoperative Prediction of Central Lymph Node Metastasis

**DOI:** 10.3389/fendo.2021.556851

**Published:** 2021-03-15

**Authors:** Sijie Chen, Chengcheng Niu, Qinghai Peng, Kui Tang

**Affiliations:** ^1^ Department of Ultrasound Diagnosis, The Second Xiangya Hospital, Central South University, Changsha, China; ^2^ Research Center of Ultrasonography, The Second Xiangya Hospital, Central South University, Changsha, China

**Keywords:** contrast enhanced ultrasound, papillary thyroid carcinoma, conventional ultrasound, Hashimoto’s thyroiditis, central lymph node metastasis (CLNM)

## Abstract

The purpose of this study was to evaluate the usefulness of the sonographic characteristics of papillary thyroid carcinoma (PTC) with Hashimoto’s thyroiditis (HT) for predicting central lymph node metastasis (CLNM). One hundred thirty-three patients who underwent thyroidectomy and central cervical lymph node dissection for PTC with coexistent HT were retrospectively analyzed. All PTCs with HT were preoperatively evaluated by ultrasound (US) regarding their nodular number, size, component, shape, margin, echogenicity, calcification, capsule contact with protrusion, vascularity and contrast enhanced ultrasound (CEUS) parameters. Univariate analysis demonstrated that patients with PTCs with HT and CLNM more frequently had age ≤ 45 years, size > 10 mm, a wider than tall shape, microcalcification, hypo-enhancement and peak intensity index < 1 than those without CLNM (all *p*<0.05). Binary logistic regression analysis demonstrated that size > 10 mm and CEUS hypo-enhancement were independent characteristics for the presence of CLNM. Our study indicated that preoperative US characteristics could offer help in predicting CLNM in PTCs with coexistent HT.

## Introduction

Papillary thyroid carcinoma (PTC) is the most common type of thyroid malignant tumor with a significantly increased incidence in recent years (1–3). Although PTC has an indolent course with a low mortality rate and satisfactory long-term prognosis, cervical lymph node metastasis remains an important factor associated with local recurrence and distant metastasis (4–7). Zaydfudim et al. identified 30,504 PTC patients (49% > 45 years old) and 2,584 follicular carcinoma patients (55% > 45 years old). The study by these researchers found that cervical lymph node metastases conferred an increased risk of death in all patients with follicular carcinoma and in those patients with PTC who were > 45 years old (8). A retrospective analysis of 399 patients demonstrated that prophylactic central compartment (level VI) neck dissection (CCND) improved disease-free survival in patients with intermediate- and high-risk differentiated thyroid carcinoma (9).

According to the American Thyroid Association’s (ATA’s) guidelines, PTC patients with clinically involved central lymph nodes (CLNs) are strongly recommended to undergo therapeutic CCND, while PTC patients with clinically uninvolved CLNs can be considered for prophylactic CCND (10). However, prophylactic CCND can result in overtreatment and lead to nerve injury to the voice, permanent hypoparathyroidism and airway function compromise (7).

Preoperative neck US for the cervical lymph nodes is recommended to evaluate the range of surgery, especially for lymph node dissection according to the ATA’s guidelines (10). However, approximately 90% of CLNMs might not be found preoperatively (11), since the sensitivity of conventional US for CLNM is less than 50% (4, 12–14). Thus, it is urgently important to identify the risk factors for PTCs with CLNM to design individualized surgical treatment strategies to avoid unnecessary prophylactic lymph node dissection.

Hashimoto’s thyroiditis (HT) is one of the most common autoimmune thyroid diseases. HT can cause the destruction of the thyrocytes by lymphocytic infiltration and interstitial fibrosis, and it results in thyroid nodules with atypical sonographic features for overlapping morphologies, margins, echogenicities and internal bloodstreams between benign and malignant lesions against an HT background. It also induces lymphadenopathy in the central compartment, which can increase the difficulty of judging metastatic lymph nodes by sonography (15).

Some authors have evaluated the biological behavior of PTC with CLNM associated with US features (16–19). In addition the widespread application of conventional B-mode US as the basis of examination, the additional use of contrast-enhanced ultrasound (CEUS) could elucidate the micro-vasculature and improve the diagnostic accuracy of thyroid nodules (20–23). However, to the best of our knowledge, the capability of CEUS for predicting CLNM in PTCs with coexistent HT has been only rarely reported. In this study, we studied the clinical characteristics and conventional US and CEUS features of PTCs with coexistent HT with or without CLNM and explored the ability to predict CLNM in PTCs against an HT background.

## Materials and Methods

### Patients

The study was approved by the ethics committee of the Second Xiangya Hospital of Central South University in China and was performed in accordance with the Declaration of Helsinki for human studies. From May 2016 to December 2018, 177 consecutive patients who had been preoperatively examined using conventional US and CEUS and had undergone surgery were enrolled in this retrospective study. The inclusion criteria were as follows: (i) pathological examination confirming PTCs with HT after surgery; (ii) patients who underwent conventional US and CEUS examinations.

**Figure 1:**
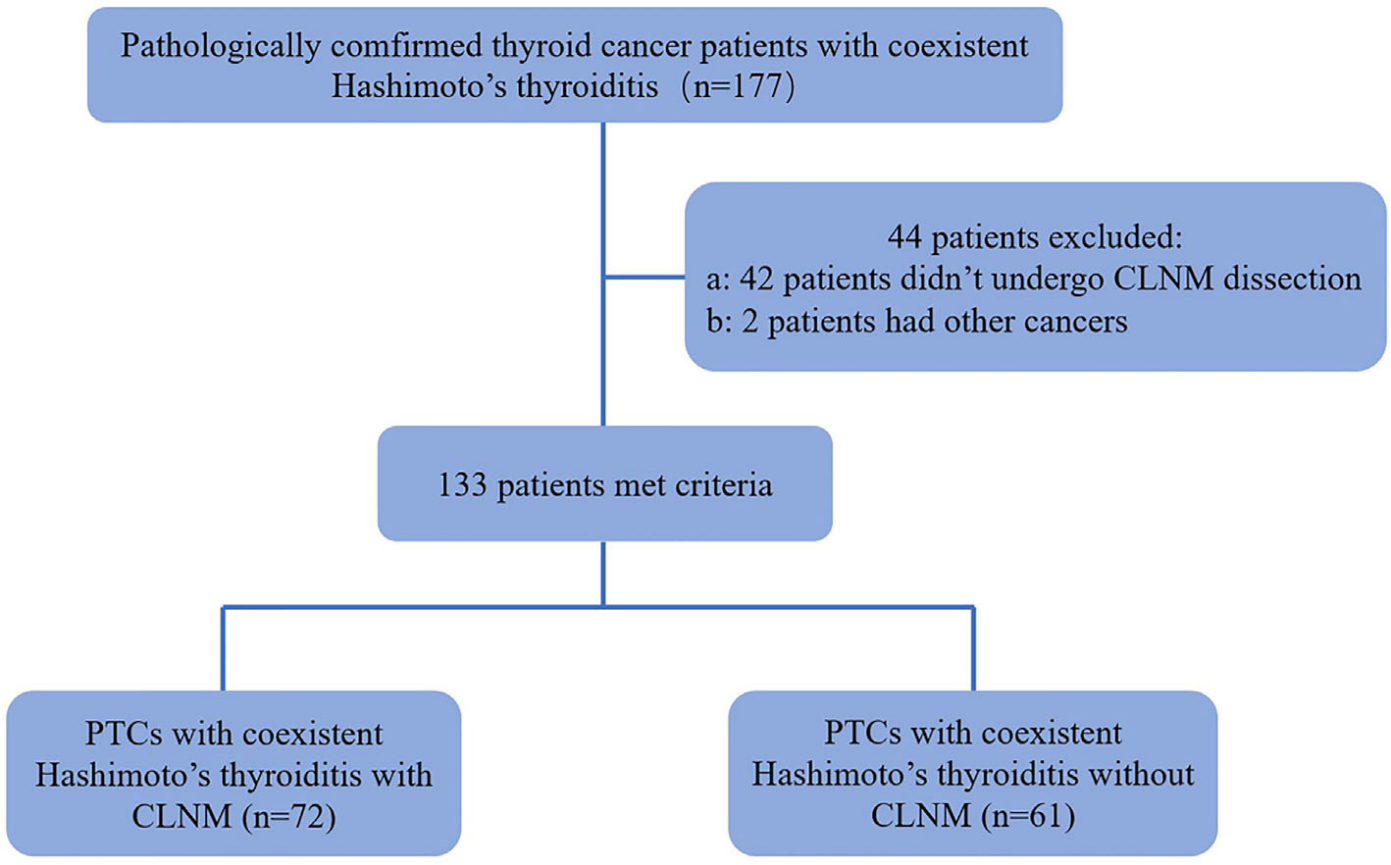
Flowchart for the selection of thyroid cancer patients. PTC, papillary thyroid carcinoma; CLNM, Central Lymph Node Metastasis.

Forty-two patients were excluded because they did not undergo central lymph node dissection. Two patients were excluded because they had different types of thyroid cancers: 1 medullary thyroid carcinoma and 1 follicular cancer. In patients with multifocal PTCs, only the largest was selected. In addition, thyroid-stimulating hormone (TSH), free thyroxine, free triiodothyronine, thyroid peroxidase antibody (A-TPO) and thyroglobulin antibody (A-TG) were measured in all of the patients before pathological examination. Thus, 133 patients (19 men and 114 women, age mean: 40.92 ± 11.21 y, range: 18-74 y) with 133 PTCs were ultimately included (**Figure 1**).

### Conventional US and Color Doppler US

All of the images in our study were acquired with Siemens Acuson S3000 US scanner (Siemens Medical Solutions, Mountain View, CA, USA) equipped with a 9L4 linear array transducer (4-9 MHz) for conventional US and CEUS. All of the selected thyroid nodules were evaluated for the following US features: size (largest diameter); composition (solid or mixed); shape (taller than wide or wider than tall); margins (well-defined or ill-defined); echogenicity (marked hypo-echoic, hypo-echoic, iso-echoic or hyper-echoic); calcification (no calcification, microcalcification<1 mm in diameter, macrocalcification >1 mm in diameter; the presence of both microcalcification and macrocalcification was defined as microcalcification); capsule contact with protrusion (present or absent); halo sign (present or absent); and internal vascularity (present or absent).

### CEUS and Analysis

Contrast pulsed sequencing (CPS) technology and dedicated analysis software (Contrast Dynamics, Mountain View, CA, USA) were used for CEUS. Three-milliliter SonoVue microbubbles (Bracco, Italy) were injected intravenously, followed by a saline flush of 5 mL. The thyroid nodule imaging lasted 60 s; the time intensity curves (TICs) within selected regions of interest (ROIs) were acquired. CEUS features of the thyroid nodules were classified as follows: enhancement type, compared with surrounding thyroid parenchyma enhancement (hyper-enhancement, iso-enhancement or hypo-enhancement); enhancement uniformity (heterogeneous enhancement or homogeneous enhancement); perfusion pattern (centripetal perfusion, the perfusion of microbubbles from the periphery to the center; centrifugal perfusion, the perfusion of microbubbles from the center to the periphery); surrounding ring enhancement, with the surrounding of the nodule revealing a ring enhancement (hyper ring enhancement, hypo ring enhancement or no ring enhancement); peak intensity (PI, expressed as a percentage); time to peak (TP, expressed in seconds); area under the curve (AUC, expressed as a percentage by seconds); and washout time (WT, expressed in seconds). PI index, TP index, AUC index and WT index are reported as indices by the ratio of the ROIs of nodules to the ROIs of thyroid parenchymal tissue.

### Statistical Analysis

SPSS software (SPSS, Chicago, IL, USA), version 21.0, was used for statistical analyses. The mean ± standard deviation (SD) was used to express continuous data, and the independent t-test was used to compare them. Percentages were used to express categorical data, and the χ^2^test were used to analyze them. Binary logistic regression was used to assess independent factors in PTCs with coexistent HT and CLNM. *p*<0.05 indicated significant differences.

## Results

A total of 133 patients with PTCs with coexistent HT, 72 (54.1%) with CLNM and 61 (45.9%) without CLNM, were included in the analysis. The clinical characteristics of the patients are shown in **Table 1**. One hundred fourteen (85.7%) PTC patients with HT were female, and 19 (14.3%) were male. Male patients constituted 16.7% of patients with CLNM and 11.5% of patients without CLNM (*p* = 0.394). The average age of PTC patients with HT with or without CLNM was 38.29 ± 10.78 years (range: 18-66 years) or 44.03 ± 10.99 years (range: 23-74 years), respectively. Fifty-three (73.6%) patients with CLNM were younger than 45 years, while only 29 (47.5%) patients without CLNM were younger than 45 years (*p* = 0.002), showing that the patients with CLNM were more inclined to be younger. Twenty-six (36.1%) patients with CLNM had multifocal cancers, and 21 (34.4%) patients without CLNM had multifocal cancers (*p* = 0.839). Forty-nine (68.1%) HT patients with CLNM had increased A-TG levels, and 55 (76.4%) had increased A-TPO levels, whereas 44 (72.1%) HT patients without CLNM had increased A-TG levels, and 42 (68.9%) had increased A-TPO levels (*p* = 0.610 and *p* = 0.330).

**Table 1:** Clinical characteristics of PTC patients with Hashimoto’s thyroiditis based on CLNM.

Characteristics	CLNM		*P* Value
	Yes (n=72)	No (n=61)	
Sex			0.394
Male Female	12 (16.7) 60 (83.3)	7 (11.5) 54 (88.5)	
Age (years) ≤ 45 y > 45 y	38.29 ± 10.78 53 (73.6) 19 (26.4)	44.03 ± 10.99 29 (47.5) 32 (52.5)	0.003* 0.002*
Multifocality			0.839
Yes No	26 (36.1) 46 (63.9)	21 (34.4) 40 (65.6)	
TSH			0.968
Normal Increased Decreased	59 (81.9) 9 (12.5) 4 (5.6)	51 (83.6) 7 (11.5) 3 (4.9)	
A-TG			0.610
Normal Increased	23 (31.9) 49 (68.1)	17 (27.9) 44 (72.1)	
A-TPO			0.330
Normal Increased	17 (23.6) 55 (76.4)	19 (31.1) 42 (68.9)	

CLNM, Central Lymph Node Metastasis; TSH, thyroid-stimulating hormone; A-TG, thyroglobulin antibody; A-TPO, thyroid peroxidase antibody.

*p < 0.05 was considered a significant difference.

**Figure 2:**
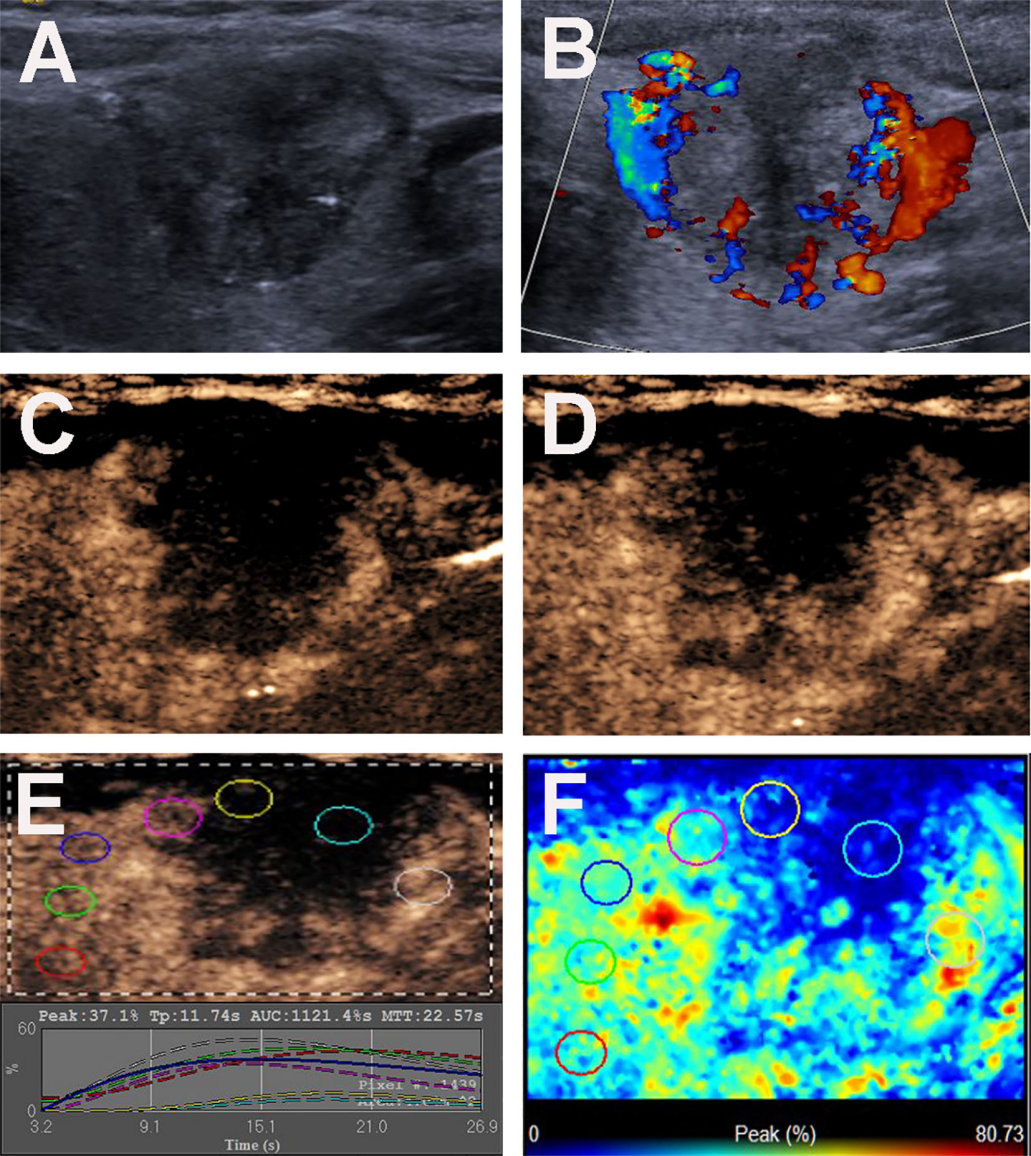
Conventional US and CEUS images of a 36-y-old female PTC patient with Hashimoto’s thyroiditis of the left thyroid lobe with CLNM. **(A)** B-mode US image revealing an 18.6-mm solid thyroid nodule with hypo-echogenicity, ill-defined margins, microcalcification, capsule contact with protrusion and halo sign. **(B)** Doppler image revealing abundant peripheral and slight internal vascularity. **(C, D)** CEUS images revealing a centripetal heterogeneous hypo-enhancement with a surrounding incomplete ring of thyroid nodule from the periphery to the center, at **(C)** 8s (wash-in) and **(D)** 13s (time to peak). **(E)** TICs of the thyroid nodule and peripheral thyroid parenchyma with different ROIs (different color circles). **(F)** Parametric color map indicating the PI values for the center of nodule was lower than those of the periphery of nodule and adjacent thyroid parenchyma.

**Figure 3:**
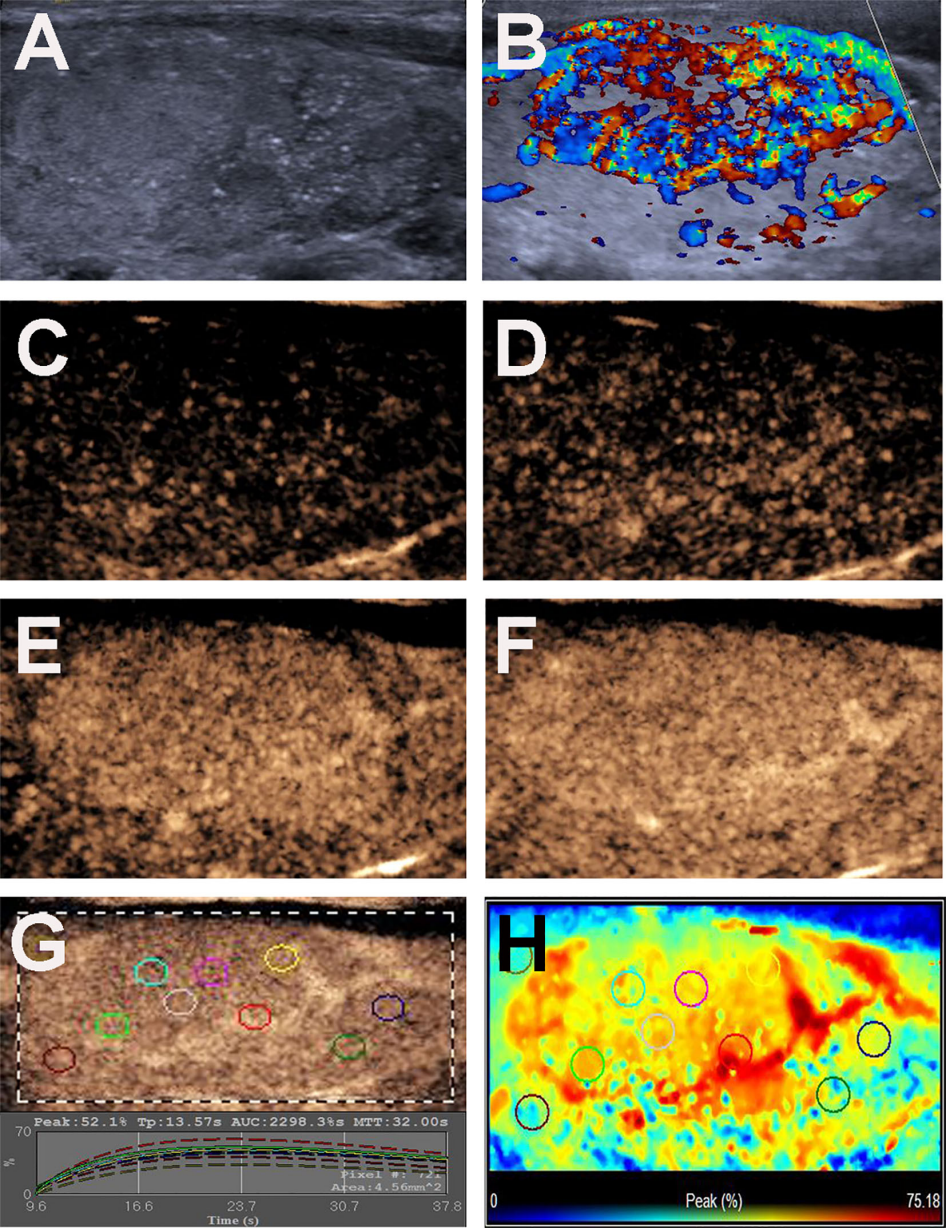
Conventional US and CEUS images of a 31-y-old female PTC patient with Hashimoto’s thyroiditis of the right thyroid lobe with CLNM. **(A)** B-mode US image revealing a 35.0-mm solid thyroid nodule with iso-echogenicity, ill-defined margins and microcalcification. **(B)** Doppler image revealing abundant peripheral and internal vascularity. **(C–F)** CEUS images revealing a centripetal homogeneous hyperenhancement with an incomplete ring of thyroid nodule from the periphery to the center, at **(C)** 11s (wash-in), **(D)** 12s, **(E)** 14s and **(F)** 17s (time to peak). **(G)** TICs of the thyroid nodule and peripheral thyroid parenchyma with different ROIs (different color circles). **(H)** Parametric color map indicating the PI values for the center of nodule was lower than that of the periphery of nodule, but higher than that of adjacent thyroid parenchyma.

The US features of the patients are reported in **Table 2**. In patients with multifocal PTCs, only the largest was selected. The mean diameters of PTCs with or without CLNM were 16.03 ± 7.62 mm (range: 6-40 mm) and 10.77 ± 5.14 mm (range: 4-26 mm), respectively, and the mean diameters of the former were significantly larger than those of the latter (*p* = 0.000). Fifty-two (72.2%) patients with CLNM had size > 10 mm, while only 24 (39.3%) patients without CLNM had size > 10 mm (*p* = 0.000). For PTCs with coexistent HT with or without CLNM, all of the nodules were solid in this study. In the PTCs with CLNM group, 60 (83.3%) the nodules had ill-defined margins (**Figure 2**), 52 (72.2%) nodules exhibited hypoechoic echogenicity (**Figure 2**), 5 (7.0%) nodules exhibited isoechoic or hyperechoic echogenicity (**Figure 3**), 62 (86.1%) nodules had microcalcification (**Figures 2** and **3**), 20 (27.8%) nodules had capsule contact with protrusion (**Figure 2**), 17 (23.6%) had the hypoechoic halo sign (**Figure 2**), and 23 (31.9%) nodules had internal blood flow (**Figures 2** and **3**). For CEUS parameters, 56 (77.8%) nodules exhibited hypo-enhancement (**Figure 2**), and 16 (22.2%) nodules exhibited hyper or iso-enhancement (**Figure 3**), indicating that the majority of the nodules underwent lower enhancement than those in parenchymal tissue. Fifty-one (70.8%) nodules showed heterogeneous enhancement (**Figure 2**), and 21 (29.2%) nodules showed homogeneous enhancement (**Figure 3**), indicating that most of the nodules received an ununiform perfusion of microbubbles. Sixty-five (90.3%) nodules had the centripetal perfusion pattern (**Figures 2** and **3**), representing most of the nodules receiving the perfusion of microbubbles from the periphery to the center. Six (8.3%) nodules existed hyper-ring enhancement (**Figures 2** and **3**). The quantitative CEUS parameters showed that 14 (19.4%) nodules had a PI index ≥1 (**Figure 3**), indicating that 19.4% of nodules had a higher PI than those of parenchymal tissue. Ten (13.9%) nodules had an AUC index ≥1 (**Figure 3**), representing that 13.9% of nodules had a larger AUC than those of the parenchymal tissue.

**Table 2:** Ultrasonographic nodule characteristics of PTC patients coexisted with Hashimoto’s thyroiditis based on CLNM.

Characteristics	CLNM		*P* Value
	Yes (n=72)	No (n=61)	
**Conventional US parameters**			
Size (mm) >10 mm ≤10 mm	16.03 ± 7.62 52 (72.2) 20 (27.8)	10.77 ± 5.14 24 (39.3) 37 (60.7)	0.000* 0.000*
Shape			0.019*
Taller than wide	13 (18.1)	22 (36.1)	
Wider than tall	59 (81.9)	39 (63.9)	
Margin			0.653
Well-defined	12 (16.7)	12 (19.7)	
Ill-defined	60 (83.3)	49 (80.3)	
Echogenicity			0.060
Marked hypoechoic	15 (20.8)	9 (14.8)	
Hypoechoic	52 (72.2)	52 (85.2)	
Iso- or hyperechoic	5 (7.0)	0 (0.0)	
Calcification			0.046*
Absent or macrocalcification	10 (13.9)	17 (27.9)	
Microcalcification	62 (86.1)	44 (72.1)	
Capsule contact with protrusion			0.070
Yes No	20 (27.8) 52 (72.3)	9 (14.8) 52 (85.2)	
Halo sign			0.199
Yes No	17 (23.6) 55 (76.4)	9 (14.8) 52 (85.2)	
Internal vascularity			0.610
Yes	23 (31.9)	17 (27.9)	
No	49 (68.1)	44 (72.1)	
**CEUS parameters**			
Enhancement type			0.012*
Hyper- or iso-enhancement Hypo-enhancement	16 (22.2) 56 (77.8)	26 (42.6) 35 (57.4)	
Enhancement uniformity			0.869
Heterogeneous enhancement Homogeneous enhancement	51 (70.8) 21 (29.2)	44 (72.1) 17 (27.9)	
Perfusion pattern			0.538
Centripetal Centrifugal	65 (90.3) 7 (9.7)	53 (86.9) 8 (13.1)	
Surrounding ring enhancement			0.549
Hyper ring enhancement Hypo ring enhancement No ring enhancement	6 (8.3) 2 (2.8) 64 (88.9)	4 (6.6) 4 (6.6) 53 (86.8)	
PI index			0.011*
≥ 1 < 1	14 (19.4) 58 (80.6)	24 (39.3) 37 (60.7)	
TP index			0.760
≥ 1 < 1	64 (90.3) 8 (9.7)	56 (91.8) 5 (8.2)	
AUC index			0.074
≥ 1 < 1	10 (13.9) 62 (86.1)	16 (26.2) 45 (73.8)	
WT index			0.947
≥ 1 < 1	8 (11.1) 64 (88.9)	7 (11.5) 54 (88.5)	

PI, peak intensity; TP, time to peak; AUC, area under the curve; WT, washout time.

**Figure 4:**
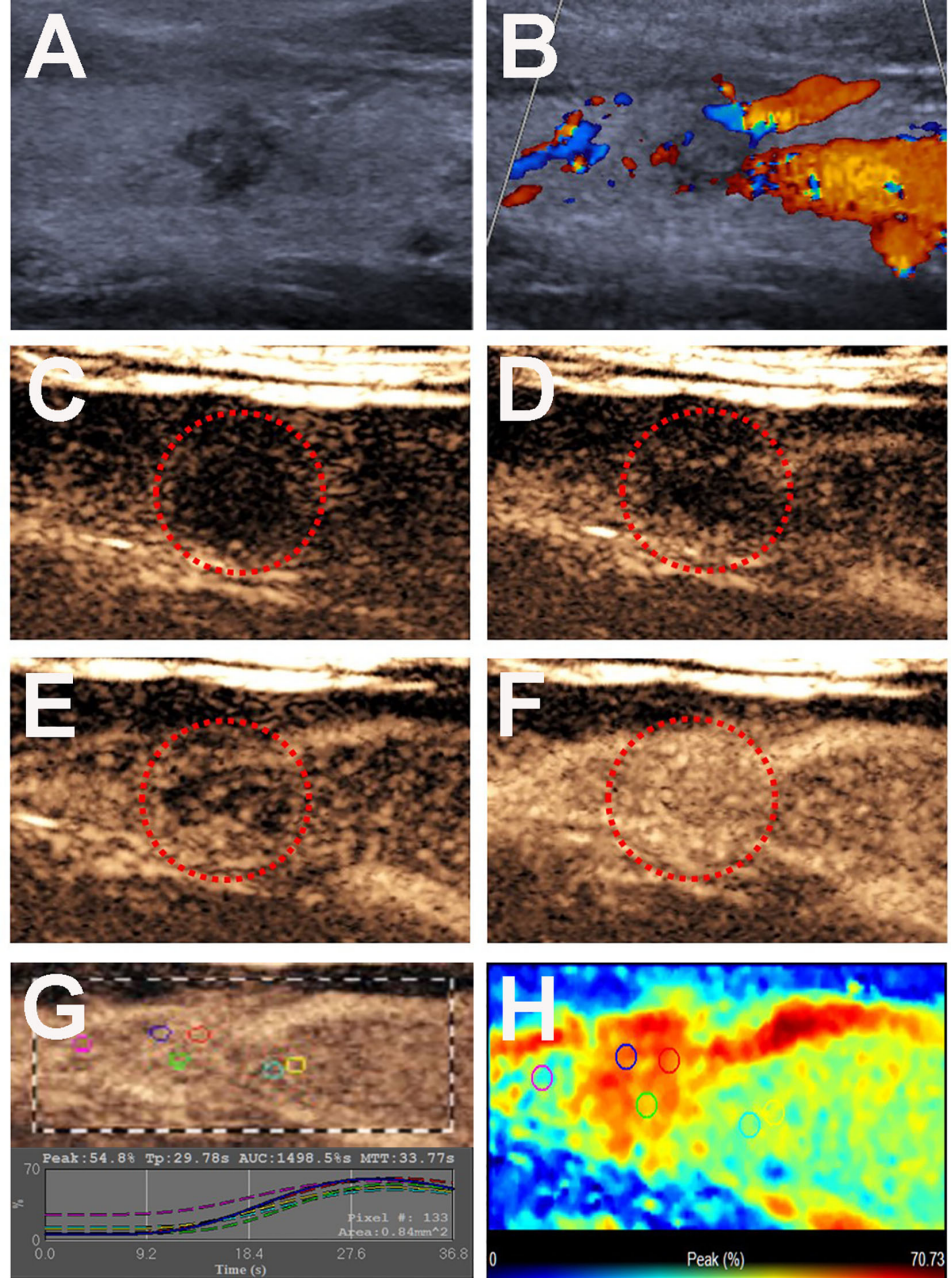
Conventional US and CEUS images of a 26-y-old male PTC patient with Hashimoto’s thyroiditis of the right thyroid lobe without CLNM. **(A)** B-mode US image revealing a 8.0-mm solid thyroid nodule with a taller than wider shape, hypo-echogenicity, ill-defined margins. **(B)** Doppler image revealing abundant peripheral vascularity and no obvious internal vascularity. **(C–F)** CEUS images revealing a centripetal homogeneous hyperenhancement of thyroid nodule from the periphery to the center, at **(C)** 2s, **(D)** 20s (wash-in), **(E)** 22s and **(F)** 34s (time to peak). **(G)** TICs of the thyroid nodule and peripheral thyroid parenchyma with different ROIs (different color circles). **(H)** Parametric color map indicating the PI values for the nodule was higher than that of adjacent thyroid parenchyma.

*p < 0.05 was considered a significant difference.

n the PTCs without CLNM group, 22 (36.1%) nodules had taller than wider shapes (**Figure 4**), 49 (80.3%) nodules had ill-defined margins (**Figure 4**), and 52 (85.2%) nodules exhibited hypoechoic echogenicity (**Figure 4**). For CEUS parameters, 26 (42.6%) nodules exhibited hyper- or iso-enhancement (**Figure 4**), 17 (27.9%) nodules showed homogeneous enhancement (**Figure 4**), 53 (86.9%) nodules had the centripetal perfusion pattern (**Figure 4**), and 53 (86.8%) showed no ring enhancement (**Figure 4**). The quantitative CEUS parameters showed that 24 (39.3%) nodules had a PI index ≥1 (**Figure 4**), 56 (91.8%) nodules had a TP index ≥1 (**Figure 4**), and 16 (26.2%) nodules had an AUC index ≥1 (**Figure 4**). Univariate analyses indicated that PTCs with coexistent HT with CLNM more often had size > 10 mm, wider than taller shape, microcalcification, hypo-enhancement and peak intensity indices < 1 compared those without CLNM (all *p* < 0.05).

Binary logistic regression analysis was performed on all of the ultrasonographic statistically significant variables (*p* < 0.05). The results indicated that size > 10 mm (B= 1.460, OR = 4.306, 95% CI = 2.239-8.278, p=0.000) and CEUS hypo-enhancement (B = 1.064, OR =2.899, 95% CI = 1.457-5.767, p=0.002) were independent characteristics for the presence of CLNM (**Table 3**).

**Table 3:** Multivariate logistic regression analysis of association of CLNMs and US characteristics in PTC patients with Hashimoto’s thyroiditis.

Parameter	B	SE	Odds ratio	95% CI	*P*
Size > 10 mm	1.460	0.334	4.306	2.239-8.278	0.000*
Hypo-enhancement	1.064	0.351	2.899	1.457-5.767	0.002*

*p < 0.05 was considered a significant difference.

## Discussion

High-resolution US is a popular method for the preoperative diagnosis of PTC and the evaluation of cervical lymph nodes. Previous studies have reported that PTC with CLNM is associated with a poor prognosis; however, not all CLNMs can be detected by US preoperatively (6, 11).

In the present study, univariate analysis showed that age ≤ 45 years was a clinical risk factor associated with CLNM in PTC patients with coexistent HT, showing that patients with CLNM were more inclined to be young people. Similar findings have been reported by other authors (17, 24, 25).

Recently, a few studies showed that US features were helpful for predicting CLNM in PTC patients. Kim et al. found that size >1 cm and markedly hypoechoic echogenicity were identified to be prognostic factors predicting CLNM (18). Nam et al. retrospectively reviewed 488 patients who underwent surgery for PTC. Malignant-looking PTCs (M-PTCs) more frequently had LN metastasis, extrathyroidal extension, and a higher stage than benign-looking PTCs (B-PTCs), especially when tumor size > 1 cm (19). In our study, we found that the tumor size > 10 mm was one of the predictive factors for CLNM.

For malignant nodules, among the US features, microcalcification, internal flow, capsule contact and involvement were identified to be independent prognostic factors for predicting CLNM (17). Subsequently, researchers found that tumor size < 10 mm was also an independent factor for CLNM in PTCs (4). In this study, on univariate analysis, we found that a wider than taller shape and microcalcification were significantly associated with CLNM of PTCs with HT, and the former perhaps contributed to the size of thyroid nodules. In this study of 22 PTCs with taller than wider shape in the non-CLNM group, 21 nodules had a size ≤ 10 mm, and the other nodule had a size > 10 mm and < 20 mm, indicating that smaller-sized thyroid nodules were more inclined to exhibit the taller than wider shape of PTCs in HT patients. Regarding microcalcification, as one of the predictive factors for CLNM, as also reported by other studies (17, 25), was exhibited in 62 (86.1%) PTCs with CLNM and 44 (72.1%) PTCs without CLNM in this study.

CEUS detects more tumor microvessels than the color Doppler techniques. Some studies have reported that CEUS could improve the diagnostic accuracy of thyroid nodes (22, 23). However, few studies have reported the association of CEUS characteristics with the predicting of CLNM in PTC patients, especially with coexistent HT. In a study by Huang et al., hyper- or iso-enhancement was predictive of the presence of CLNM (25). Hyper- or iso-enhancement suggests that the tumor blood supply is greater or equal to the surrounding thyroid parenchyma. Hypo-enhancement indicates that the tumor blood supply is less than in the surrounding thyroid parenchyma. However, in our study, 42 (31.6%) PTCs with HT showed hyper- or iso-enhancing parametric maps, while only 16 (38.1%) of these patients presented with CLNM, which was significantly less than in HT patients with PTCs without CLNM (61.9%). The results indicated that hypo-enhancement in PTC patients with HT was considerably more frequent in the CLNM group than in the non-CLNM group, inconsistent with the study of Hong et al. (25). Similarly, the peak intensity index was significant lower in HT patients with PTCs with CLNM than that of HT patients with PTCs without CLNM. Due to the destruction of thyrocytes by lymphocytic infiltration and interstitial fibrosis in HT disease, the thyroid parenchyma displayed changes in echogenicity and blood supply; the abundant blood supply in the thyroid parenchyma could strengthen the parenchymal enhancement in CEUS mode and bring out a lower nodule enhancement by contrast, which was variable for the degrees of lymphocytic infiltration and interstitial fibrosis. To the best of our knowledge, these CEUS parameters for predicting CLNM in PTCs with coexistent HT have not been explored to date. According to the results of binary logistic regression analysis, tumor size >10 mm and CEUS hypo-enhancement were independent characteristics for the presence of CLNM in PTC patients with coexistent HT. If a young PTC patient has a thyroid tumor size > 10 mm and CEUS hypo-enhancement preoperatively, prophylactic CCND is suggested according to this study.

This study had several limitations. First, only patients with pathological results were enrolled, some of the PTC patients did not undergo surgery, and some of the PTCs subjected to thyroidectomy without central lymph node dissection were missed; thus, selection bias was present. Second, the nodule size differences in this study were enormous, which might have affected the US characteristics of PTCs. A large-scale and prospective multicenter study is needed to clarify these findings.

## Conclusions

In HT patients, age ≤ 45 years, size > 10 mm, wider than tall shape, microcalcification, hypo-enhancement and peak intensity index <1 in the preoperative US and CEUS findings were significantly associated with CLNM of PTCs. Multivariate analysis demonstrated that tumor size > 10 mm and CEUS hypo-enhancement were independent characteristics for the presence of CLNM. Thus, preoperative US characteristics could be helpful in predicting CLNM in PTC patients with HT.

## Data Availability Statement 

The raw data supporting the conclusions of this article will be made available by the authors, without undue reservation.

## Ethics Statement

The studies involving human participants were reviewed and approved by the ethics committee of the Second Xiangya Hospital of Central South University in China. The patients/participants provided their written informed consent to participate in this study. Written informed consent was obtained from the individual(s) for the publication of any potentially identifiable images or data included in this article.

## Author Contributions

CN contributed to the conception and design of the work. SC and CN participated to data analysis and manuscript writing. QP and KT participated to data collection and patients’ follow-up. All authors contributed to the article and approved the submitted version.

## Funding

This project was funded by the National Natural Science Foundation of China (Grant No. 81974267), Hunan Provincial Natural Science Foundation of China (Grant 2018JJ2575) and Hunan Provincial Health Commission Research Foundation Project (B2019166).

## Conflict of Interest

The authors declare that the research was conducted in the absence of any commercial or financial relationships that could be construed as a potential conflict of interest.
